# Four guidelines on cardiovascular complications and cardiovascular disease, prognosis and treatment in COVID‑19

**DOI:** 10.1007/s12471-021-01570-x

**Published:** 2021-04-15

**Authors:** H. J. Siebelink, C. W. Jansen, E. Belfroid, J. Hoogervorst-Schilp

**Affiliations:** 1 Department of Cardiology, Leiden University Medical Center, Leiden, The Netherlands; 2 Netherlands Society of Cardiology, Utrecht, The Netherlands; 3 Dutch Association of Medical Specialists, Utrecht, The Netherlands

This supplement of the *Netherlands Heart Journal* contains four guidelines on COVID-19 for patients with cardiovascular risk factors or cardiovascular disease. In March 2020, when the COVID-19 pandemic reached the Netherlands, the Dutch Cardiovascular Alliance (DCVA) launched the idea to set up a COVID-19 registry to collect data and learn more about this new threat to human health. In the DCVA a number of organisations involved in cardiovascular research cooperate on topics ranging from basic science to implementation of cardiovascular healthcare. The CAPACITY registry was set up for patients admitted to hospital with COVID-19 and aimed to collect data on cardiovascular outcome as well as comorbidity and the results of clinical investigations. CAPACITY is closely connected to the ISARIC WHO registry on COVID-19. In August 2020, approximately 40% (*n* = 5524) of the Dutch COVID-19 patients admitted to hospital were included in CAPACITY through registration in more than 50 centres all over the Netherlands. The CAPACITY registry focusses on several topics such as cardiovascular risk factors, cardiac damage, prognosis and the influence of medication [1].

Apart from data registration, healthcare professionals realised that information on how to manage our patients was lacking or inadequate. Therefore, the Netherlands Society of Cardiology (NVVC) addressed the need for clinical guidelines on COVID-19 patients with cardiovascular risk factors or cardiovascular disease. Together with the Dutch Association of Medical Specialists (Federatie Medisch Specialisten), and with the support of the Dutch Society of Intensive Care, Netherlands Association for Cardiothoracic Surgery and the patient organisation Heart Counsel, four guidelines were developed. The subjects of these four guidelines are:

1. How often and to what extent do admitted COVID-19 patients have signs of cardiac injury?
2. Impact of cardiovascular risk factors and cardiovascular disease in hospitalised COVID-19 patients
3. The effects of ACE2 expression mediating pharmacotherapy in COVID-19 patients
4. Effect of anticoagulant therapy in COVID-19 patients

The guidelines were developed by a guideline committee of professionals (see Appendix 1 for members) and the Knowledge Institute of Medical Specialists (KIMS). The project started in June 2020 and was completed in February 2021; the procedure for the development and format of the guidelines is described below.

## General format

The guideline was drafted in accordance with the requirements outlined in the ‘Guidelines 2.0’ report of the Guideline Advisory Committee of the Council on Science, Education and Quality [2]. This report is based on the AGREE II instrument (Appraisal of Guidelines for Research & Evaluation II) (www.agreecollaboration.org), an instrument designed to assess the quality of guidelines with broad international support.

As a first step in the development, all relevant stakeholders were consulted to define key issues that should be addressed in the guideline. The guideline committee prioritised the issues and formulated clinical questions according to the PICO system (Patient, Intervention, Control, Outcome). Outcome measures that were relevant for the patient for each primary question were determined.

## Literature search, results and grading

Systematic literature reviews were performed. Specific search terms were used to identify published scientific studies related to each individual primary question in Medline and Embase. Members of the guideline committee selected articles identified by the search based on predetermined criteria. The selected articles were used to answer the clinical question. The search strategies are listed for each individual primary question in the online supplements. Individual studies were assessed systematically, based on predefined methodological quality criteria in order to assess the risk of biased study results. These assessments can be found the evidence tables on quality assessment or risk of bias in the Electronic Supplementary Material for each module.

The relevant study results from all selected articles were presented in evidence tables. The key findings from the literature are described in the literature summary. If studies were sufficiently similar in design, data were also summarised quantitatively (meta-analysis) using Review Manager 5. The level of scientific evidence was determined using the GRADE method. GRADE is short for ‘Grading Recommendations Assessment, Development and Evaluation’ (see http://www.gradeworkinggroup.org/) [3]. Conclusions were drawn based on the body of evidence.

## Recommendation

To formulate clinical recommendations, scientific evidence was considered together with other key aspects, including patient preferences, costs, availability of facilities and/or organisational aspects and the (preliminary) results from the CAPACITY registry [4]. Based on the balance between favourable and unfavourable effects for the patient following the evidence synthesis, together with the additional arguments, recommendations were formulated. These recommendations provide an answer to the clinical question. The level of scientific evidence and the importance given to considerations by the guideline committee jointly determine the strength of the recommendation. In accordance with the GRADE method, a low level of evidence for conclusions in the systematic literature review does not rule out a strong recommendation, while a high level of evidence may be accompanied by weak recommendations. The strength of the recommendation is always determined by weighing all the relevant arguments.

## Commentary and authorisation phase

After reaching consensus in the guideline committee, the draft guideline was subjected to peer review by all the relevant stakeholders. The guideline committee adjusted and finalised the draft guideline based on the comments. The final guideline was submitted for authorisation to the (scientific) organisations involved and authorised by them.

## Conclusion and perspective

The four guidelines described in this supplement are the result of the most urgent clinical questions from healthcare professionals posed in the beginning of the COVID-19 pandemic. With the third wave of COVID-19 about to hit Dutch society, the four questions remain relevant. We realise that there are many more questions to answer in order to best treat our patients. These four questions, however, had the priority at the start of this guideline process and we expect that the answers will be of help to manage our patients.

Relevant other questions on prevalence of cardiac damage caused by COVID-19, treatment and long-term outcome are now defined as gaps in the evidence and prioritised in the multidisciplinary ‘Kennisagenda’ COVID-19 from the Dutch Association of Medical Specialists (https://www.demedischspecialist.nl/sites/default/files/Kennisagenda%20COVID-19.pdf).

Research funder ZonMw has already announced that the knowledge gap in cardiology can be addressed in a new call for COVID-19 research. We hope that in the near future professionals will work together to answer these, and many other questions, in order to treat and improve the health of our patients and reduce the burden of COVID-19 on society.

## Appendix

### Members of the guideline committee

- H.J. (Hans-Marc) Siebelink, cardiologist, Leiden University Medical Center, Leiden; NVVC (chair)
- F.W. (Folkert) Asselbergs, cardiologist, University Medical Center Utrecht, Utrecht; NVVC
- P.C. (Peter) Smits, cardiologist, Maasstad Hospital, Rotterdam; NVVC
- L.S.D. (Lucia) Jewbali, intensive care cardiologist, Erasmus Medical Center, Rotterdam; NVVC and NVIC
- Y.M. (Yigal) Pinto, cardiologist, Amsterdam University Medical Center, Amsterdam; NVVC
- R.G. (Robert) Tieleman, cardiologist, Martini Hospital, Groningen; NVVC
- J. (Jeroen) Schaap, cardiologist, Amphia Hospital, Breda; NVVC
- P. (Pim) van der Harst, cardiologist, University Medical Center Utrecht, Utrecht; NVVC
- R. (Roland) van Kimmenade, cardiologist, Radboud University Medical Center, Nijmegen; NVVC
- R.A. (René) Tio, cardiologist, Catharina Hospital, Eindhoven; NVVC
- F.A. (Erik) Klok, internist vascular medicine, Leiden University Medical Center, Leiden; Netherlands Association of Internal Medicine (NIV)
- C.M.H.B. (Carolien) Lucas, cardiologist, Alrijne Hospital, Leiderdorp and Alphen aan de Rijn; NVVC
- I. (Inge) Schalkers, patient-participation policy advisor, Heart Counsel (Harteraad)

### NVVC

- C.W. (Karin) Jansen, senior policy advisor, NVVC
- D.G. (Debby) Keuken, senior policy advisor, NVVC

### Knowledge Institute of Medical Specialists (KIMS)

- J. (Janneke) Hoogervorst-Schilp, advisor, Knowledge Institute of Medical Specialists (KIMS), Utrecht
- E. (Evelien) Belfroid, advisor, Knowledge Institute of Medical Specialists (KIMS), Utrecht
